# Comparison of a Micro-Neutralization Test with the Rapid Fluorescent Focus Inhibition Test for Measuring Rabies Virus Neutralizing Antibodies

**DOI:** 10.3390/tropicalmed2030024

**Published:** 2017-07-07

**Authors:** Todd G. Smith, Amy T. Gilbert

**Affiliations:** 1 Poxvirus and Rabies Branch, Division of High-Consequence Pathogens and Pathology, Centers for Disease Control and Prevention, 1600 Clifton Road Northeast, Atlanta, GA 30329, USA; 2 National Wildlife Research Center, US Department of Agriculture, Animal and Plant Health Inspection Service, Wildlife Services, 4101 LaPorte Avenue, Fort Collins, CO 80521, USA; Amy.T.Gilbert@aphis.usda.gov

**Keywords:** rabies, virus neutralizing antibodies, diagnostic test, assay development, assay validation

## Abstract

The rapid fluorescent focus inhibition test (RFFIT) is routinely used in the United States to measure rabies virus neutralizing antibodies (rVNA). RFFIT has a long history of reproducible and reliable results. The test has been modified over the years to use smaller volumes of reagents and samples, but requires a 50 μL minimum volume of test serum. To conduct pathogenesis studies, small laboratory animals such as mice are regularly tested for rVNA, but the minimum volume for a standard RFFIT may be impossible to obtain, particularly in scenarios of repeated sampling. To address this problem, a micro-neutralization test was developed previously. In the current study, the micro-neutralization test was compared to the RFFIT using 129 mouse serum samples from rabies vaccine studies. Using a cut-off value of 0.1 IU/mL, the sensitivity, specificity, and concordance of the micro-neutralization test were 100%, 97.5%, and 98%, respectively. The geometric mean titer of all samples above the cut-off was 2.0 IU/mL using RFFIT and 3.4 IU/mL using the micro-neutralization test, indicating that titers determined using the micro-neutralization test are not equivalent to RFFIT titers. Based on four rVNA-positive hamster serum samples, the intra-assay coefficient of variability was 24% and inter-assay coefficient of variability was 30.4%. These results support continued use of the micro-neutralization test to determine rabies virus neutralizing antibody titers for low-volume serum samples.

## 1. Introduction

Measurement of rabies virus neutralizing antibody (rVNA) is essential to evaluating pre- or post-exposure prophylaxis and rabies diagnosis in humans and vaccination status in domestic animals [1,2,3]. The rapid fluorescent focus inhibition test (RFFIT) is one WHO-recommended test for measuring rVNA [3]. The RFFIT is used widely, primarily in the US, due to the standardized and functional results produced [4]. Developed in 1973 as a replacement for the mouse neutralization test, the RFFIT represented a major advance in cost, time, and replacement of animal use [5]. When compared to the mouse neutralization test, the RFFIT was 95% concordant, 100% sensitive, and 83% specific [5]. Over the years, the RFFIT method has been modified to use mouse neuroblastoma cells in place of BHK cells [6], to use a 96-well format similar to the tissue culture serum neutralization test or more widely used fluorescent antibody virus neutralization (FAVN) test [7,8,9], and to use half the volume of reagents. Despite these modifications, the RFFIT method requires a minimum volume of 50 μL of serum per test [10].

Kuzmin, et al. (2008) developed a micro-neutralization test based on the RFFIT to measure rVNA in serum samples with limited volume, e.g., from bats [11]. The micro-neutralization test has numerous advantages compared to the standard RFFIT, including the need for only 3 μL of serum per test. Additionally, instead of using 8-well, chamber slides, the micro-neutralization test uses 4-well, Teflon-coated slides, which decreases the reagent content by ~90% representing a cost savings compared to the traditional RFFIT. Also, the dilutions are simplified starting with 10^−1^, and only 10 fields in each well are scored for results rather than 20 fields, representing a time saving compared to the RFFIT. Furthermore, because the first dilution is higher than RFFIT, the micro-neutralization test is less susceptible to cytotoxicity. Overall, the micro-neutralization test is less labor intensive than the Terasaki plate method [12], but similar to the RFFIT, the micro-neutralization test still requires a 20–40 h incubation period, highly skilled personnel, and appropriate biocontainment and biosafety risk mitigation associated with use of live rabies virus.

The objective of the current study was to compare the rVNA measured in the same test serum using the RFFIT and micro-neutralization test. Mouse serum generated for other rabies immunization experiments was curated for volume and quality. The sample set was run with both tests using the same lots of reagents and rabies virus strain CVS-11. Results were compared based on sensitivity and specificity.

## 2. Materials and Methods

Approved animal use protocols were established with CDC’s Institutional Animal Care and Use Committee (protocols 2330SMIMOUC, 2332SMIMOUC, 2622SMIHAMC). For mouse serum, adult female CD-1 mice were purchased from Charles River Laboratories (Wilmington, MA, USA). All animals received experimental or commercial rabies vaccine on day 1. Approximately 0.2 mL of blood was collected using the submandibular technique on days 0, 15, and 30. Serum was separated and stored at ≤−10 °C. After the primary studies were completed, a convenient set of 129 serum samples were selected based on sufficient volume, previous RFFIT result (positive or negative), and lack of cytotoxicity. Cytotoxic samples were excluded because they could not be accurately classified as positive or negative in the RFFIT. Of the convenient sample, 55 had detectable rVNA (>0.05 IU/mL) and 74 had no detectable rVNA (<0.05 IU/mL) by RFFIT.

For hamster serum, adult female LVG Syrian hamsters were purchased from Charles River Laboratories (Wilmington, MA, USA). All animals were challenged with rabies virus on day 0. Some animals received post-exposure prophylaxis with commercial human rabies vaccine and human rabies immune globulin or experimental monoclonal antibody product on day 1, followed by additional doses of vaccine on day 4 and 8, while some animals received no post-exposure prophylaxis. Approximately 0.2 mL of blood was collected using the subclavicle technique on days 0, 4, 8 and at termination. Serum was separated and stored at ≤−10 °C. After the primary studies were completed, four serum samples were selected to determine assay variability.

The RFFIT was completed according to a standard protocol [10]. The micro-neutralization test was modified slightly from the previous report [11]. The test was set up in a humidity chamber made from a petri dish and wet paper towel to prevent evaporation from the wells. An amount of 3 μL of test serum or standard rabies immune globulin (SRIG, US FDA lot R-3) was serially diluted in 12 μL of Dulbecco’s minimal essential medium supplemented with 10% fetal bovine serum in each well of a Teflon-coated, 4-well slide. An amount of 12 μL of rabies virus CVS-11 (CDC lot V-404) diluted to 50 FFD_50_/mL (50 × 50% fluorescing foci doses/mL) was added to each well. Back titration of the rabies virus CVS-11 and cell-only control were completed in a separate 4-well slide. Slides were incubated 60 min at 37 °C with 0.5% CO_2_. 24 μL of mouse neuroblastoma cells diluted to approximately 5 × 10^5^ cells/mL was added to each well and slides were incubated 20 h at 37 °C with 0.5% CO_2_. Slides were fixed with acetone for 30 min at −20 °C and stained with FITC-anti-rabies virus antibodies (Fujirebio Diagnostics, Malvern, PA, USA) with additional 0.001% Evan’s Blue for 30 min at 37 °C with 0.5% CO_2_. Slides were washed twice with 0.1 M PBS pH 7.5 and observed with a fluorescence microscope. In each well, 10 fields at 20× magnification were scored based on presence/absence of fluorescent foci and endpoint titer was calculated using the Reed-Muench method. Endpoint titers were converted to international units per milliliter (IU/mL) based on comparison to the SRIG diluted at 2 IU/mL.

Results for the same sample from two independent tests were compared. The cut-off of 0.1 IU/mL was chosen empirically based on approximately 50% neutralization in the first dilution of the micro-neutralization test. The same cut-off was chosen for RFFIT based on equivalency which is approximately complete neutralization in the first dilution. Sensitivity, specificity, predictive values, and 95% confidence intervals were calculated using GraphPad Prism v6. The correlation between quantitative titers determined by either test was evaluated using the Pearson test with α = 0.05. Coefficient of variability was calculated by dividing the standard deviation in IU/mL by the mean in IU/mL.

## 3. Results

Based on the 0.1 IU/mL cut-off, 127 (98%) of 129 mouse serum samples tested had concordant results between the two tests. The remaining two samples were above the cut-off based on RFFIT but fell below the cut-off in the micro-neutralization test (Table 1). This results in a sensitivity of 100% (CI = 92.6–100%) and specificity of 97.5% (CI = 91.4–99.7%) for the micro-neutralization test. The positive predictive value was 96% (CI = 86.3–99.5%), the negative predictive value was 100% (CI = 95.4–100%), and the false negative rate for the new test was 2.5%.

**Figure 1:**
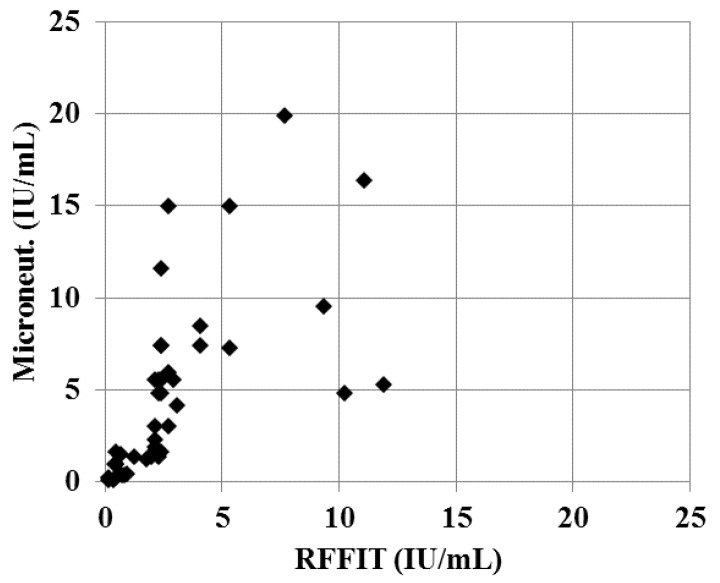
Comparison of rVNA titers (IU/mL) measured by the micro-neutralization test (microneut.) and RFFIT. Rabies virus neutralizing antibody was measure in individual serum samples by the micro-neutralization test (Microneut.) and RFFIT. Positive rVNA titers in IU/mL were plotted on a linear scale. The axis limits exclude four samples that had very high rVNA titers by both tests. The geometric mean titer was 3.4 IU/mL with the micro-neutralization test compared to 2.0 IU/mL with the RFFIT.

Using the micro-neutralization test, 37% of samples were positive for rVNA compared to 39% with RFFIT. While the number of positive samples in a set may be slightly underestimated, the individual titers of positive samples appear higher using the micro-neutralization test (Figure 1). The geometric mean titer of rVNA positive samples was 3.4 IU/mL with the micro-neutralization test and 2.0 IU/mL with the RFFIT. Because of this difference in the individual rVNA titers, the correlation between the two tests was poor (R^2^ = 0.55).

Intra- and inter-assay coefficient of variability was calculated using IU/mL from six statistical replicates, from two biologic replicates, for four rVNA-positive hamster serum samples. The intra-assay coefficient of variability ranged from 21–24% for the statistical replicates, and the median inter-assay coefficient of variability was 30.4% for the biologic replicates.

## 4. Discussion

A rabies virus micro-neutralization test is necessary to measure rVNA from small volume samples. The method described herein has been developed over time to meet this need. For purposes of this study, only serum from vaccinated animals was used, which is different, in terms of immune profile, from serum collected from wildlife to estimate prevalence of rabies virus natural infection/exposure. The above comparison to the RFFIT demonstrates that the method has utility for measuring rVNA in samples from vaccinated animals. This will be extremely beneficial in small animal challenge studies validating efficacy of medical countermeasures, where serum volumes are often limited.

Additional validation of the micro-neutralization test for study of natural rabies virus exposure in different taxa, geographic regions, and divergent lyssaviruses is still an important consideration. For purposes of validation, this study would fall under stage one: comparison to a standard test method [13]. Based on the sensitivity and specificity calculated here, 2% error, and 95% confidence, a panel of 95 rVNA positive samples, ideally from naturally infected/exposed animals, and 279 rVNA negative samples from the same species, would be required to validate the diagnostic sensitivity and specificity of the assay [13].

Endpoint titers, including for SRIG, were higher in the micro-neutralization test than RFFIT. Paradoxically, the micro-neutralization titers in IU/mL were also higher, meaning the endpoint titers of test serum increased more than the endpoint titer of SRIG. This trend holds true even when a subset of 20 samples with rVNA <1 IU/mL (RFFIT) is analyzed; although the difference is less for this subset. Because of the difference in individual titers, results from the micro-neutralization test should not be aggregated with results from RFFIT. The differences in titers may be due to differences in the evaporation, contact surface area, or fluid dynamics in the small drop used in the micro-neutralization test. Using a humidity chamber as described in the methods section is recommended to prevent evaporation especially when a large number of samples are being tested. In the future, the method could be further optimized to address the differences in the titers.

The 0.1 IU/mL cut-off provided high levels of agreement between the micro-neutralization test and RFFIT. Using a higher or lower cut-off of 0.05 IU/mL or 0.5 IU/mL resulted in 95% and 93% concordance, respectively, compared to 98% concordance at 0.1 IU/mL. Based on these results, the 0.1 IU/mL cut-off is recommended for determining positive and negative rVNA in the micro-neutralization test.

The coefficient of variability shows that the micro-neutralization test is consistent and reliable for measuring rVNA. The variability is consistent with published variability for the RFFIT of 18–26% intra-assay and 28–30% inter-assay [4]. Given the advantages of the micro-neutralization test including sample/reagent sparing and time/labor saving, this assay should be used when the RFFIT is not feasible. Overall, the favorable comparison between the micro-neutralization test and RFFIT supports its continued use for measuring rVNA in small-volume samples.

## Figures and Tables

**Table 1:** Sensitivity, specificity, as well as positive and negative predictive value of the micro-neutralization test compared to the rapid fluorescent focus inhibition test (RFFIT).

Test	Microneut. ^1^ ≥0.1 IU/mL	Microneut. <0.1 IU/mL	Total	PPV NPV ^2^
**RFFIT ≥0.1 IU/mL**	48	2	50	96%
**RFFIT <0.1 IU/mL**	0	79	79	100%
**Total**	48	81	129	
**SN/SP ^3^**	100%	97.5%		

^1^ Micro-neutralization test; ^2^ Positive predictive value (PPV), Negative predictive value (NPV); ^3^ Sensitivity (SN), Specificity (SP).
